# Low Cost Electrode Assembly for EEG Recordings in Mice

**DOI:** 10.3389/fnins.2017.00629

**Published:** 2017-11-14

**Authors:** Emily C. Vogler, Daniel T. Flynn, Federico Busciglio, Ryan C. Bohannan, Alison Tran, Matthew Mahavongtrakul, Jorge A. Busciglio

**Affiliations:** ^1^Institute for Memory Impairment and Neurological Disorders, University of California, Irvine, Irvine, CA, United States; ^2^School of Education, University of California, Irvine, Irvine, CA, United States; ^3^Dodge College of Film and Media Arts, Chapman University, Orange, CA, United States; ^4^Department of Neurobiology and Behavior, University of California, Irvine, Irvine, CA, United States

**Keywords:** electroencephalography, EEG, electrode implantation, wireless electroencephalography, neurologger

## Abstract

Wireless electroencephalography (EEG) of small animal subjects typically utilizes miniaturized EEG devices which require a robust recording and electrode assembly that remains in place while also being well-tolerated by the animal so as not to impair the ability of the animal to perform normal living activities or experimental tasks. We developed simple and fast electrode assembly and method of electrode implantation using electrode wires and wire-wrap technology that provides both higher survival and success rates in obtaining recordings from the electrodes than methods using screws as electrodes. The new wire method results in a 51% improvement in the number of electrodes that successfully record EEG signal. Also, the electrode assembly remains affixed and provides EEG signal for at least a month after implantation. Screws often serve as recording electrodes, which require either drilling holes into the skull to insert screws or affixing screws to the surface of the skull with adhesive. Drilling holes large enough to insert screws can be invasive and damaging to brain tissue, using adhesives may interfere with conductance and result in a poor signal, and soldering screws to wire leads results in fragile connections. The methods presented in this article provide a robust implant that is minimally invasive and has a significantly higher success rate of electrode implantation. In addition, the implant remains affixed and produces good recordings for over a month, while using economical, easily obtained materials and skills readily available in most animal research laboratories.

## Introduction

The use of animals to model human disease pathology has required the development of technology to investigate the effects of experimental interventions in subjects for which existing equipment designed for imaging, recording, or measuring physiology of humans are inappropriate due to the differences between humans and animals in size, other physical attributes, and compliance with equipment requirements. One such physiological recording is electroencephalography (EEG), the recording of changes in electrical potentials at the surface of the brain through scalp or skull, which are a result of ion flow across neural membranes and a measure of neuronal activity (Petsche et al., 1984). EEG is commonly used in the clinical study and diagnosis of medical disorders, and with the advent of computer quantitative analysis, is also used to quantify the effects of pharmacological, dietary, or genetic alterations in research studies (Shipton, 1975; Bronzino, 1984).

The process for EEG in humans involves attaching electrodes directly to the scalp with an adhesive or wearing a cap with the electrodes attached, with wires connecting the electrodes to the recording equipment. This process benefits from a compliant subject and limits mobility and so is difficult to use in experiments requiring awake and mobile animal subjects. Advances in miniaturization of recording equipment have resulted in wireless EEG recording devices that can be implanted in the animal or mounted on the animal's head, providing mobility for the animal during recording and allowing recording for hours and even days (Higashi et al., 1979). Such devices incorporate either telemetry or data saved to a microchip and typically require inserting screws into holes drilled into the skull, affixing screws with cyanoacrylate glue to the surface of the skull, or attaching a pre-fabricated headmount with screws and dental cement, which require lengthy and invasive surgical procedures and costly materials. The screws or headmounts are frequently connected to wires by soldering or glue, which can form fragile connections or interfere with conductance of the electrical signal.

In our initial experiments using the Neurologger device (TSE Systems) to record EEG in mice, we used the protocol and electrode material provided by TSE Systems, which calls for soldering and screws, either inserted in drilled holes or glued on the surface of the skull. The TSE protocol also calls for removal and re-insertion of the pins of the screw leads into the connecting block while the animal is under anesthesia, which increases the risk of damage to the soldered connections and results in a lengthy surgical procedure. Using this protocol, we experienced a significant failure rate, typically failed connectivity between neural tissue and recording device with one or more electrodes, and decreased tolerability of the lengthy surgery in aged mice.

Consequently, we developed a new method that combines fabrication of a simple wiring harness with insulated silver-plated copper wire electrodes, which eliminated the screws, solder, and glue in the electrode assembly and eliminated the process of re-inserting the pins into the connector during surgery. This method results in electrodes with direct contact of brain tissue and minimal headmount apparatus. The headmount is well-tolerated and will remain on the animal for a month or more and, most importantly, these procedures simplify the implantation process, resulting in a quick, and efficient surgery that minimizes discomfort to the animal and promotes swift recovery. The procedures were designed to utilize laboratory and surgical equipment customarily found in animal research facilities and readily available tools and materials. It greatly facilitates implementation of wireless EEG recording devices in animal research.

## Materials and methods

All procedures were approved by the Institutional Animal Care and Use Committee of the University of California, Irvine. Materials for constructing and implanting the electrode assembly are readily available from electronic supply stores or online.

### Preparing the components for the electrode connector assembly (embedded link to Video 1)

**Figure 1:**
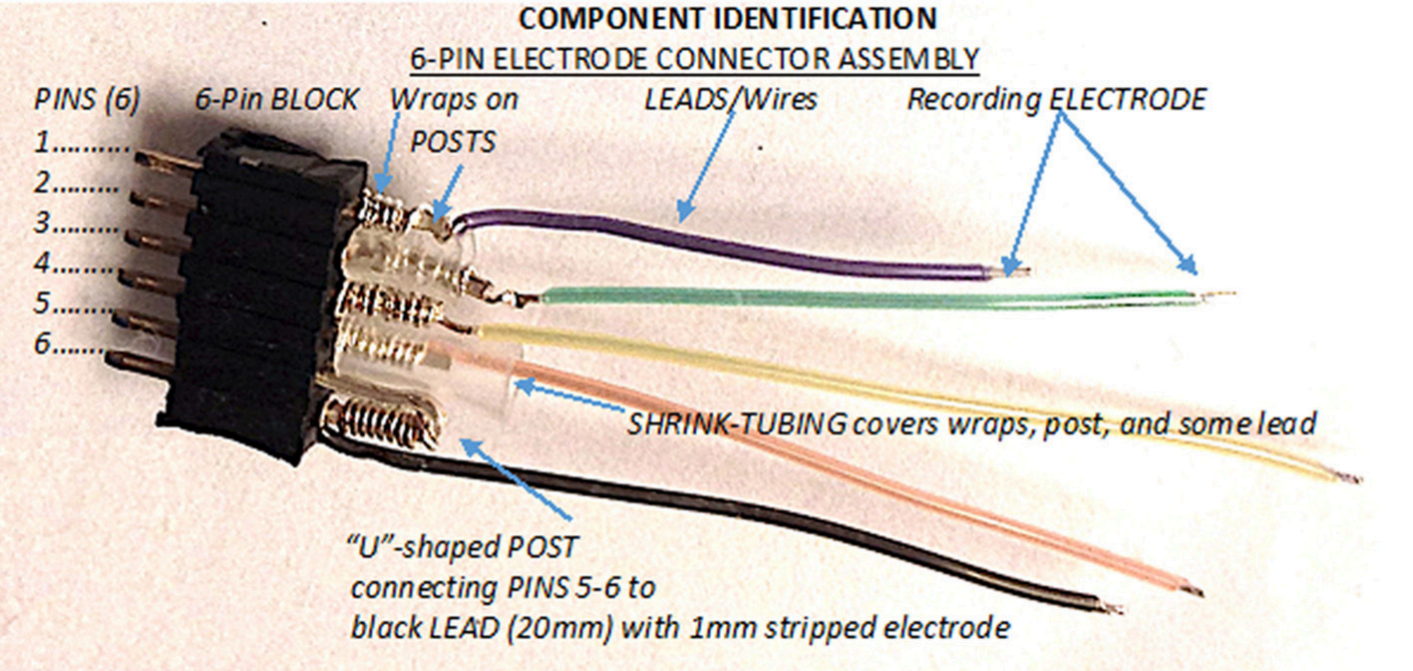
The three main components of the 6-pin electrode connector assembly. The 6-pin electrode connector assembly is comprised of the 6-pin block, the posts, and the recording leads/wires, 1 of which serves as the ground-reference electrode and 4 that serve as the ADC electrodes.

The electrode assembly has three main components: the 6-pin connector block, the posts that connect the block to the leads, and the leads with the recording electrodes at their tips (Figure 1). The first step is to make the 6-pin connector block from the supplied 25-pin connector block. To do this, count out 7 pins, giving you one sacrificial pin, and then cut between the 7th and 8th pin of the larger 25-pin connector block. Trim the excess pin and material so you have 6 pins comprising one 6-pin block.

Then prepare posts, which are created from the legs of standard 3 mm LEDs. The diameter of each LED leg is 0.5 mm and fits securely in the holes of the pin block. Use sand paper or an abrasive sponge to clean the oxidation from the LED's legs. An electronic designer breadboard holds the LEDs and components for the construction process. Cut the LED component off as scrap, leaving two 20 mm-long LED legs to be used as posts to connect the leads into the 6-pin connector block. After creating the posts, the next step is to secure the lead-to-post connection using the “wire-wrap” process. There are 2 separate types of leads. One lead will serve as the ground and reference lead and will connect to 2 pins in the connector block. The remaining 4 leads are ADC or signal leads and each connect individually to a single post and single pin in the connector block.

### Building the ground-reference lead (embedded link to Video 2)

Making the ground and reference lead, which has 1 lead for 2 pin connections, requires only 1 post made from 1 LED leg. Fully insert 1 post into the designer's breadboard. To wire-wrap the lead wire to the post, strip a 25 mm length of black insulated wire and insert the stripped section into the small hole at the outer-edge of the wire wrap tool. With the stripped section of the wire fully inserted into the edge hole of the tool, place the center hole of the wire wrap tool completely over the post held in the breadboard until the tool rests on the breadboard. Holding the insulated section of the wire onto the breadboard, slowly twist the tool to wrap the stripped section of wire securely around the post. When done accurately, the wraps will be neat and close together and there will be no insulated wire wrapped around the post.

Unique to the ground and reference lead, the connection is to 2 pins of the 6-pin block. Thus, the single post is bent into a tight “U” with the wrap at the apex of the curve. To accomplish this, hold the post with needle-nose pliers at the wraps. Then bend the post into a tight “U.” After bending into the tight “U,” the black lead is now functionally connected to two parallel lengths of post which, when trimmed to “pin-length,” will be inserted into holes 5 and 6 in the pin connector. Pinch the “U” close together so it matches the width of the receiving holes in the 6-pin block. For the pins and block to connect properly, ~3 mm of post is exposed beyond the wraps. This is approximately the same length as the pins that are extending from the opposite side of pin block. Insert the 2 posts of the “U” into the last 2 holes in the 6-pin block corresponding to pins 5 and 6. Make sure to insert the posts solidly into the pin block. Check for continuity between pins 5 and 6 using a digital multi-meter to insure a mechanical connection between the lead wire and both pins after insertion.

Shrink tubing on the exposed lead at the wire wrap provides 2 things: (1) Electrical insulation from neighboring wrapped posts, and (2) Strain-relief for the wire lead. Cut a small (~5 mm) piece of shrink tubing—just enough to cover the exposed wraps and a small section of insulated wire. Slide the shrink tubing over the exposed lead and wrapped section of the post going into the pin block. Use a micro-torch to shrink the tubing onto the inserted post's wrap and lead. Strip the insulation from the lead to leave ~10 mm of insulated wire beyond the wrap. Just beyond the insulation at the opposite end of the lead, trim to ~0.5 mm of uninsulated lead. This will be the recording electrode that will be gently implanted into the hole drilled into the mouse's skull. Be careful to firmly hold only the lead when stripping insulation.

### Building the analog-digital channel (ADC) leads (embedded link to Video 3)

The next step is to make the 4 ADC leads. Since 4 leads are required for each assembly, prepare 4 posts from the legs of 2 LEDs. Each ADC lead will each have only 1 lead connected to 1 post to be inserted into 1 hole in the 6-pin block. It is helpful during the surgical process to differentiate ADC leads by differing insulation color. Follow the same wire wrapping procedure as before for each ADC lead. Wire wraps should be tight and evenly spaced. For ADC leads, trim and remove the post above the wire wrap, where the insulated section of wire begins, so the wraps are on the post where the wire lead's insulation begins. Trim the post below the wire wraps to create ~3 mm of exposed post below the wraps. Insert the 3 mm of exposed post into hole 4 in the pin block adjacent to the ground-reference lead in holes 5 and 6 until it is completely and securely inserted. Strip the trailing end of the lead to have ~10 mm of insulated wire extending from the wrapped post. Trim the stripped section of lead to leave only 0.5 mm of uninsulated wire at the end of the lead to serve as the recording electrode. Repeat that process for the remaining 3 ADC leads, placing each new lead into the next adjacent hole. Putting shrink tubing on every post and wrap is not possible due to size constraints. Insulating alternating post-wraps can ensure insulation and strain relief between posts. Check continuity to make sure that electrical connections between each pin of the block and the end of each lead are intact. Confirm that only the pins that are connected to the leads have continuity and that there are no shorts or connections between adjacent pins, wraps, or electrodes. Sterilize the completed assembly with a hard surface disinfectant such as Cetylcide-II per manufacturer's directions. Do not autoclave the assembly.

### Preparing for surgical implantation of electrodes (embedded link to Video 4)

Prepare and anesthetize the mouse for surgery, then place the mouse into a stereotaxic device. Lift a section of the scalp with forceps to make an incision through the raised area. Then cut a teardrop-shaped piece of scalp encompassing the electrode implantation sites, removing the scalp and any underlying tissue to expose bare skull. Swab the exposed skull with 70% ethanol to remove any remaining tissue and clean the skull. Locate bregma or any other reference point you will use to determine the stereotaxic locations. Insert a marking pen into the stereotaxic device, center the pen on bregma (or your point of choice), and use the micrometer function of the stereotaxic device and pen to locate and mark the electrode implantation sites relative to your reference point. Using a sterilized drill bit of the same diameter as the electrodes, drill holes just penetrating the skull at each marked electrode location, careful not to pass into the brain. The skull will discolor as the drill penetrates. Remove the drill if any blood appears or when there is a sudden drop in resistance as this indicates penetration of the skull. Swab skull material off the drill bit with a sterile tissue or gauze with 70% ethanol after drilling each hole. Swab off any blood so it does not coagulate and plug the hole.

### Implanting the electrodes (embedded link to Video 5)

Place the electrode assembly in the desired location over the back of the mouse, keeping the pin block parallel to the mouse's back, and use forceps to bend the electrode wires to align with the drilled holes. Load a gel-loading tip on a 20 μl pipettor with Vetbond by depressing the pipettor plunger, inserting the gel tip into the Vetbond bottle, inverting the bottle and flicking it gently to get the adhesive to settle into the tip of the bottle and then drawing the Vetbond into the gel tip with the pipettor plunger. The Vetbond will stay fluid in the tip for at least 10–15 min. Use bent toothed forceps to insert the electrode wires into the drilled holes, then secure each wire to the skull with 2–3 μl of Vetbond. Take care not to allow Vetbond to get into the mouse's eyes or flow into empty drilled holes. Keep a sterile cotton swab handy to wipe up any excess Vetbond. Repeat the insertion and securing process for the remaining electrodes.

### Affixing the electrode assembly to the mouse head (embedded link to Video 6)

Use dental cement to further secure the electrodes in the skull and to build a pedestal to support the recording device. Mix resin into the cement powder and apply the cement around all the electrodes, covering the entire exposed skull and taking care not to allow the cement to get in the subject's eyes. Keep a sterile cotton swab handy to catch any dripping cement. Position the pin connector block in the desired location and completely cover all leads between the skull and the connector block by applying subsequent layers of dental cement, creating a secure pedestal of dental cement to support, and secure the pin connector. Allow the cement to set up between layers and to prevent the formation of holes or gaps which could allow the mouse to snag and pull off the assembly. It is critical that the supporting cement places the pin connector centered on the mouse's midline and high enough that the recording apparatus will not rub the animal's back or become dislodged if the animal has a seizure as a result of anesthesia administration. Check that the dental cement has set, then secure the seam between the cement and the scalp with a thin line of Vetbond. Check that the cement and Vetbond forms a seal all around the scalp incision. Test the continuity between the reference and ground electrodes to ensure that the connections of the electrode assembly were not compromised during the implantation process. This completes the surgical process.

### Attaching the recording device

The recording device can be attached to and removed from the implanted electrode assembly without anesthesia on animals that can be held securely. Aggressive animals may need to be briefly anesthetized for attachment and removal. Subsequent recordings can be made during the course of a month or more.

## Results

Twenty-four mice were implanted with screw electrodes and 24 mice were implanted with wire electrodes. Each animal was implanted with 4 recording electrodes placed at −1.34 A/P, +1.50 L; −1.34 A/P, −1.50 L; −3.5 A/P. +3.00 L; −3.50 A/P. −3.00 L and one ground/reference electrode place at −6.00 A/P, +0.50 L relative to bregma. A recording electrode implantation was considered successful if the electrode recorded data consistent with the other electrodes and was considered unsuccessful if it showed periods with clipped signal both at minimum and maximum voltage, no change in potential and/or a 60 Hz mains hum.

**Figure 2:**
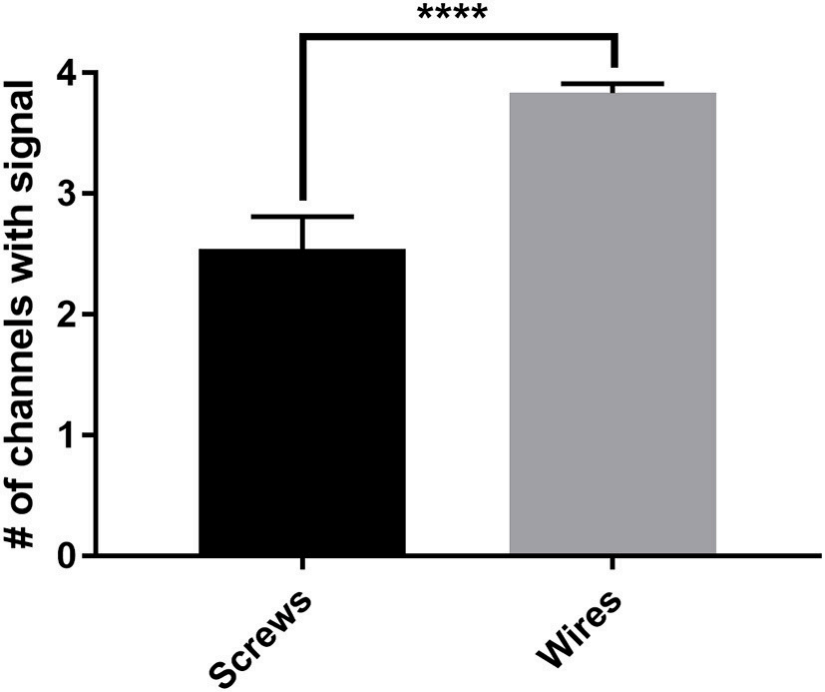
The average number of successful electrode implantations increases 51% with the wire method. The new wire electrode method resulted in an average of 3.8 ± 0.08 of the 4 recording electrodes implanted per mouse returning analyzable signal, compared to an average of 2.5 ± 0.27 of the 4 recording electrodes implanted per mouse with the screw electrode method, an increase of 51% in successful implantations as measured by individual implanted electrodes. Screws, *n* = 24 mice; wires, *n* = 24 mice, ^****^p < 0.0001. Data were analyzed by two-tailed unpaired *t*-test. Error bars indicate the mean ± SEM.

**Figure 3:**
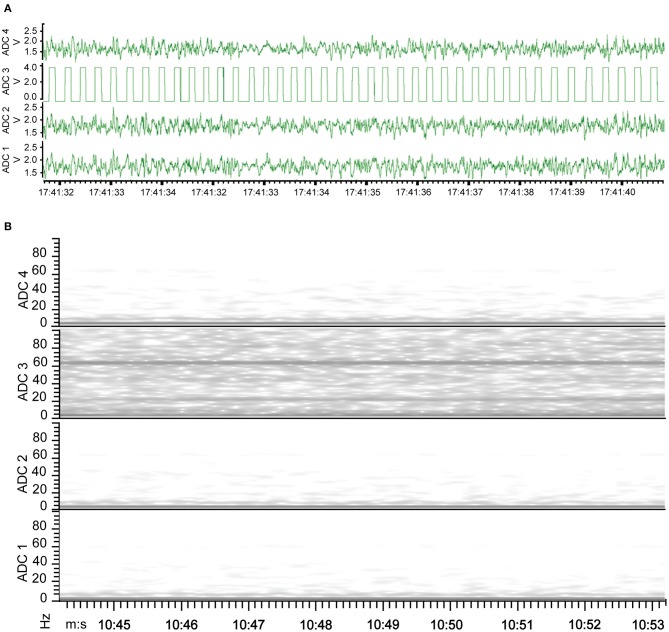
EEG of electrode implantation failure. **(A)** Waveform representation of the analog signal illustrates a clipped signal from the improperly implanted electrode of ADC2 as the recorded signal exceeded the Neurologger's EEG-specific signal magnitude capability. **(B)** Time-frequency representation of the analog signal indicates the continuity between the electrode and recording device failed with electrode of ADC2, resulting in no EEG signal recorded. Consequently, the graph has a band at 60 Hz recorded from other electrical equipment present.

The new wire method of electrode assembly and implantation resulted in a 51% increase in the number of successfully implanted electrodes, from an average of 2.5 of the 4 recording electrodes per mouse providing signal with the screw method to an average of 3.8 of the 4 recording electrodes providing signal with the wire method (Figure 2). An unsuccessful implantation typically results in a clipped signal (Figure 3A) with a 50 or 60 Hz mains hum from background electromagnetic emissions (Figure 3B).

**Figure 4:**
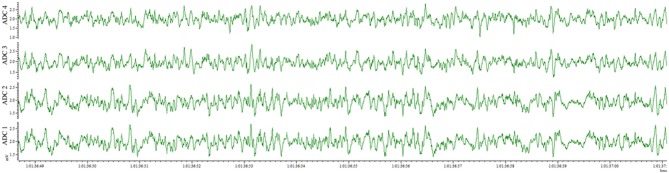
EEG recorded 4 days post-surgery utilizing the wire method. All 4 electrodes record analyzable signal.

**Figure 5:**
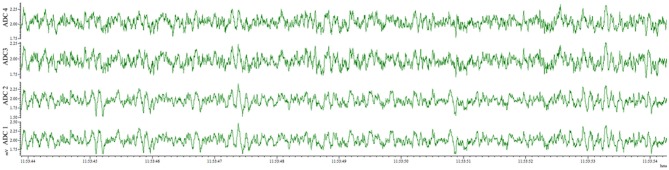
EEG recorded 28 days post-surgery utilizing the wire method. All 4 electrodes record analyzable signal, with no deterioration compared to 4 days post-surgery.

This method also results in an implantation that is well-tolerated by the animal for weeks and provides the opportunity for repeated recordings after experimental interventions. EEG traces recorded from the same animal 4 days after electrode implantation (Figure 4) and 1 month after the first recording (Figure 5) indicate no degradation of the signal over that time period, demonstrating the robustness of the electrode implantation process including the dental cement headmount. There were 4 separate 2-day recording sessions, 1 each week during the month, for a total recording time of over 200 h. The subject mouse ate, nested, and groomed normally during the course of the month and was caged individually to prevent cage mates from chewing on the assembly.

## Discussion

The use of animals in research was for many years limited to studies of diseases and pathologies of the particular species of animal or testing of pharmaceuticals for lethality or adverse effects, which has limited translation to effects on humans. However, use of animals to study disease has increased significantly in the last 30 years due to the creation of genetically modified (transgenic) animals to study gene function and to model human diseases. While many animal species have been successfully genetically modified, mice are the most commonly used species because more than 80% of human genes have corresponding counterparts in the mouse genome (Emes et al., 2003), their short life span reduces the time needed to complete age-dependent studies, and colonies are easily maintained. It is now possible to mimic pathology characteristic of a human disease to which mice are not normally susceptible, such as the neurodegenerative conditions of Alzheimer's and Parkinson's. The pathologies can then be studied both *in vivo* and *in vitro* and potential therapeutics can be administered to the animal subjects to find candidate treatments for human clinical trials. These genetic advances in research technology require the adaptation of equipment designed to quantify changes in physiology of human subjects to suit the physical and behavioral characteristics of mice, which in general requires miniaturizing while maintaining robustness of the equipment so it is not overly obstructive to the mouse's activities but can withstand damage from normal grooming activities.

The ability to record EEG in mice has led to significant research findings, including age-related sleep disturbances and changes in EEG profile (Jyoti et al., 2015) and the presence of seizure activity in mouse models of Alzheimer's Disease (AD) (Palop et al., 2007), a condition previously not often recognized in AD patients due to their cognitive deficits (Vossel et al., 2013). Just a few years ago, EEG studies of AD patients focused on sleep abnormalities (Jeong, 2004), altered regional connectivity, and rhythms (Knyazeva et al., 2013), while more recent research is also utilizing mouse models of AD to discover the underlying causes of seizure activity in AD pathology and investigate therapeutic treatments (Sanchez et al., 2012; DeVos et al., 2013; Bomben et al., 2014; Born et al., 2014).

Introducing the ability to record EEG on freely moving mice performing behavioral tasks makes it possible to observe and test interventions in mice designed to affect learning and memory that would be hampered by tethering or other invasive equipment. Typically, screws are used as electrodes or anchors for wires serving as electrodes and there are two different approaches for affixing the screws. One features drilling holes in the skull and inserting screws, which requires careful precision to avoid drilling or inserting the screws too deep and causing brain damage (Lapray et al., 2008; Armstrong et al., 2013), while the other avoids drilling and instead affixes the screws to the surface of the skull with cyanoacrylate glue (Etholm et al., 2010), which can result in poor contact if there is excessive glue between the skull and screw. After utilizing both approaches to record EEG on mice with a wireless recording system, with a lack of consistent successful implantation and adequate signal conduction, our lab decided to investigate alternative approaches and have developed procedures that result in consistent successful surgeries and recordings, with a robust implant that is minimally invasive and produces recordings for a month or more after implantation. The use of screws appeared to be the main impediment to consistent results due to the large size relative to a mouse. A flat screw base does not provide a secure conductance area on a curved mouse skull, while drilling several holes large enough for screws in the small and thin mouse skull resulted in a fragile setup that failed to maintain the apparatus for long periods of time. We also found that tissue growing around the wound with screw implants would degrade the signal within 2 weeks of electrode attachment. The other weak link in the process is the attachment of wires to the screws and pin connectors with the use of solder or conductive adhesive. These connections are difficult to manufacture and fragile in use.

**Figure 6:**
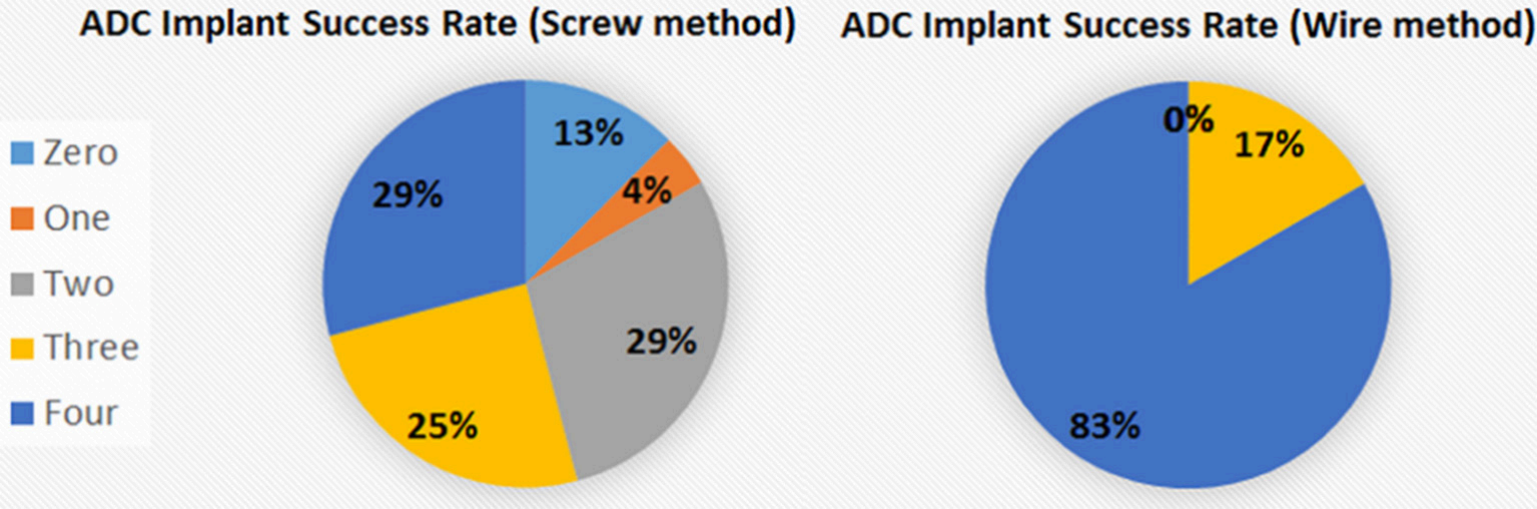
Percentage of electrodes successfully implanted. The percentage of fully successful electrode implantation, as measured by implant recording signal on all 4 ADC channels, increased from 29% with the screw method to 83% with the wire method. The different areas represent the number of electrodes recording analyzable signal after implantation for the 2 methods.

Our electronic technician suggested inserting just a small length of highly conductive stripped wire through a small hole drilled in the skull and affixing the wire jacket insulation to the skull with adhesive, with the wire wrapped around the pin connector instead of soldered to it, and proposed to make a pre-assembled wiring harness that would be strong and simple to affix. A similar process without screws has been described for telemetry recording of EEG (Weiergräber et al., 2005), but specific details were not provided. Consequently, we have completely revamped our electrodes and implantation procedures, finding that the percentage of successful recordings from all electrodes in an implanted electrode assembly increased from 29% with the TSE screw method to 83% with the new wire method (Figure 6). The success rate is calculated based on signal return from 0 through 4 electrodes as failure of the ground/reference electrode typically results in loss of signal from all recording electrodes even if the recording electrodes are all properly implanted. Other benefits of the wire method are that the implants remain in place for extended periods with no adverse effects or reactions and the assembly can be constructed with equipment readily available from typical electronic stores. On the other hand, some limitations of this method include not being well-suited for studies where more electrodes are needed to resolve different frequencies during sleep stages or for studies targeting deep brain structures which require implantation of depth electrodes.

Advances in science and technology over the past 30 years have made possible the development of transgenic animals to model human diseases and electronic equipment that can wirelessly record 72 h of EEG data on a device weighing slightly more than the U.S. Mint specifications for a dime (www.USMint.gov). However, it is still important to think “keep it simple” when using such equipment to reduce the opportunities for failure of components or steps in the process. Simplifying the electrodes and implantation process is also in accordance with the animal research principles of “replacement, reduction and refinement (Russell and Burch, 1959),” in that this is a refinement that minimizes the invasiveness of the experimental intervention and reduces the number of animal subjects needed to complete a study by increasing the reliability of data generation from each animal subject. Technology advances by both grand leaps and small adjustments; ironically, the wire-wrapping technology is well over a half century old and relatively novel to the current generation of scientists and has been documented to have the greatest reliability among various methods of electronic connections (Wagner, 1999). We have presented a protocol for improvements in the implementation of miniaturized EEG equipment that will facilitate successful use of the equipment in animal research.

## Author contributions

EV and JB designed the project. EV wrote the manuscript. EV and MM acquired and analyzed the data. MM and JB revised the manuscript. DF, MM, RB, and AT refined the technique. FB and DF recorded and edited the videos.

### Conflict of interest statement

The authors declare that the research was conducted in the absence of any commercial or financial relationships that could be construed as a potential conflict of interest.
